# Arginine 125 Is an Essential Residue for the Function of MRAP2

**DOI:** 10.3390/ijms23179853

**Published:** 2022-08-30

**Authors:** Maria Rosaria Fullone, Daniela Maftei, Martina Vincenzi, Roberta Lattanzi, Rossella Miele

**Affiliations:** 1 Department of Biochemical Sciences “Alessandro Rossi Fanelli”, Sapienza University of Rome, Piazzale Aldo Moro 5, I-00185 Rome, Italy; 2 Department of Physiology and Pharmacology “Vittorio Erspamer”, Sapienza University of Rome, Piazzale Aldo Moro 5, I-00185 Rome, Italy

**Keywords:** prokineticins, MRAP2, obesity, GPCR

## Abstract

MRAP2 is a small simple transmembrane protein arranged in a double antiparallel topology on the plasma membrane. It is expressed in the paraventricular nucleus of the hypothalamus, where it interacts with various G protein-coupled receptors, such as the prokineticin receptors, and regulates energy expenditure and appetite. The aim of this work was to analyze the functional role of the specific arginine residue at position 125 of MRAP2, which affects protein conformation, dimer formation, and PKR2 binding. Results obtained with the MRAP2 mutants R125H and R125C, which are found in human patients with extreme obesity, and mouse MRAP2, in which arginine 125 is normally replaced by histidine, were compared with those obtained with human MRAP2. Understanding the mechanism by which MRAP2 regulates G protein-coupled receptors helps in elucidating the metabolic pathways involved in metabolic dysfunction and in developing new drugs as specific targets of the MRAP2-PKR2 complex.

## 1. Introduction

Melanocortin receptor accessory protein 2 (MRAP2), a member of the MRAP protein family, is a small single transmembrane protein arranged in antiparallel dual topology at the plasma membrane [1,2]. It is expressed almost exclusively in the paraventricular nucleus of the hypothalamus, where it regulates energy expenditure and appetite. MRAP2 interacts with several G protein-coupled receptors (GPCRs), such as melanocortin, ghrelin, and orexin receptors, and influences their expression, localization, signaling, and internalization. Recently, MRAP2 has been shown to increase food intake by inhibiting prokineticin receptors (PKRs) [3,4,5]. PKRs are GPCRs expressed both centrally and peripherally. In particular, PKR2 is mainly localized in the central nervous system (CNS), whereas PKR1 is found in the peripheral tissues. PKRs couple to G_αq/11_, G_αs_, and G_αi_ after prokineticin 2 (PK2) binding, mediating several signaling pathways that promote increases in intracellular calcium and cAMP levels, Akt phosphorylation, and activation of ERK and STAT3.

Centrally, in neurons of the hypothalamic arcuate nucleus, binding of PK2 to PKR1 induces activation of STAT3 and release of melanocyte-stimulating hormone (MSH), leading to anorectic effects and modulation of thermoregulation and energy homeostasis. In contrast, PK2β, an alternative splicing short isoform of PK2 composed of only 47 amino acids, shows limited ability to induce STAT3 phosphorylation and leads to increased food intake [6,7,8]. In adipocytes, PK2 limits preadipocyte proliferation and differentiation by binding to PKR1 to control adipose tissue expansion [9,10].

Mutations of arginine 125 in histidine (R125H) or cysteine (R125C) have been detected in the conserved C-terminal region of MRAP2 in humans, where they are correlated with an extreme obesity phenotype [11,12,13]. Mouse MRPA2, despite high sequence identity with its human counterpart, normally exhibits a substitution of arginine 125 for histidine, and shows greater efficiency in modulating the activity of PKRs.

We produced two MRAP2 mutants in which the Arg125 residue was replaced by histidine or cysteine, then analyzed their interactions with PKR2 using yeast as a heterologous expression system. In addition, we examined PK2-stimulated G_αi_ signaling in CHO cell lines expressing PKR2 in the presence of the MRAP2 mutants. The C-terminal soluble domain of the MRAP2 mutant proteins was expressed in *Escherichia coli* and biochemically characterized. The aim of this work was to analyze the functional role of the specific arginine residue in position 125 of MRAP2 in affecting the protein structure, dimer formation, and conformational changes by comparing the mutants with human and mouse MRAP2.

## 2. Results

### 2.1. PKR2 Activation and Glycosylation in the Presence of Mouse MRAP2 and Human MRAP2 Mutants

**Figure 1:**
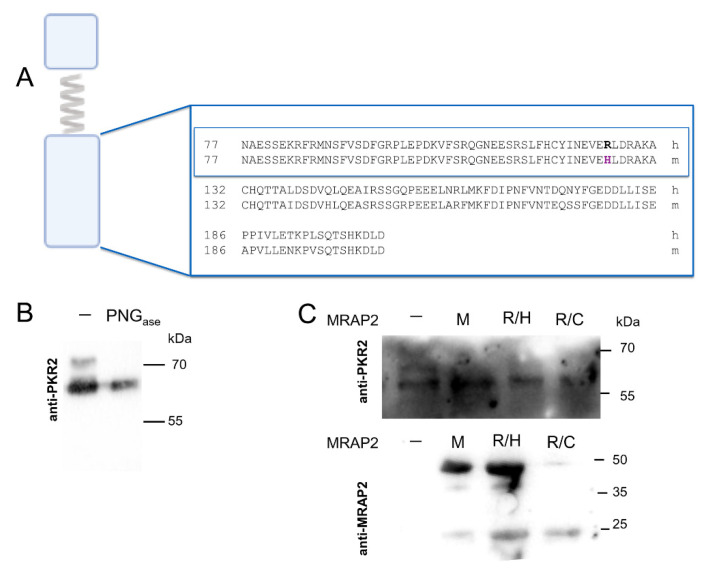
Analysis of PKR2 glycosylation in CHO cells in the presence of mouse MRAP2 and MRAP2 mutants. (**A**) Alignment of the human and mouse MRAP2 C-terminal domain. The sequence alignments of the region important for dimerization are shown in the box. (**B**) CHO stably expressing PKR2 untreated membrane proteins or those treated with PNGase F were analyzed by western blot analysis using an anti-PKR2 antibody. (**C**) Membrane proteins CHO stably expressing PKR2 were analyzed by western blot analysis using anti-PKR2 and anti-MRAP2 antibodies. In the absence of MRAP2, –; in the presence of mouse MRAP2, M; MRAP2 R125H mutant, R/H; MRAP2 R125C mutant, R/C.

The C-terminal MRAP2 domain is involved in PKR2 localization and activation. Recently, we identified a region from asparagine at position 77 to alanine at position 131 involved in specific interaction with the extracellular N-terminal sequence of PKR2 [5]. The arginine residue at position 125 is extremely conserved in primates and replaced by histidine in rodents (Figure 1A). Interestingly, mutation of arginine 125 with histidine or cysteine leads to hyperphagic obesity in humans [11,12,13].

In CHO-PKR2 cells, we examined the ability of mouse MRAP2, R125H, and R125C mutants to affect the level of glycosylation and signaling of PKR2. The results showed that glycosylation was impaired in the presence of mouse MRAP2 and the R125H and R125C mutants (Figure 1B,C). In CHO PKR2 cells, the expression levels of the MRAP2 mutants R125H and R125C are different, although in both cases they are sufficient to inhibit PKR2 receptor glycosylation.

In CHO cells stably transfected with PKR2, PK2 is able to activate ERK and this activation is abrogated by pretreatment with the G_αi_ inhibitor pertussis toxin [6]. Moreover, the effect of PK2 is significantly reduced in the presence of MRAP2 [5]. We therefore, examined ERK and STAT3 phosphorylation after PK2 incubation of CHO-PKR2 cells in the presence or absence of the MRPA2 mutants R125H and R125C. Additional experiments were performed by co-transfecting CHO-PKR2 cells with mouse MRAP2.

**Figure 2:**
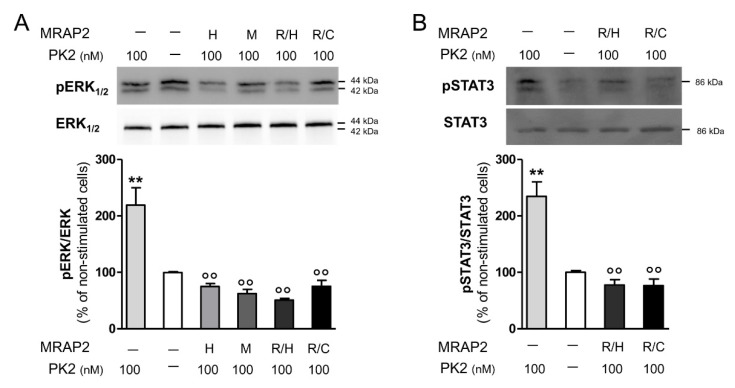
Analysis of ERK and STAT3 phosphorylation in CHO-PKR2 cells. Representative western blots and densitometric plots showing p-ERK and ERK (**A**) and p-STAT3 and STAT3 (**B**) protein levels after 10 min and 1 h treatments, respectively, with PK2 100 nM. protein levels after 10 min treatment with PK2 100 nM in presence of human MRAP2, H; mouse MRAP2, M; R125H MRAP2, R/H; R125C MRAP2, R/C. The bar graph data are presented as a ratio of pERK/ERK and plotted as the % increase with respect to non-stimulated cells. Bar plots indicate the means ± SEM obtained from the three experimental conditions. One-way ANOVA followed by Tukey’s test for multiple comparisons was used for statistical evaluation; ** *p* < 0.01 vs. non-stimulated cells, °° *p* < 0.01 vs. PK2 100 nM stimulated cells.

Both the R125H mutant and mouse MRAP2 further reduced the ERK and STAT3 phosphorylation induced by PK2. In the presence of the MRAP2 mutant R125C, the level of ERK phosphorylation was comparable to that observed in the presence of MRAP2 wt (Figure 2).

### 2.2. Analysis of the Interaction of MRAP2 Protein Variants with PKR2 in S. cerevisiae

**Figure 3:**
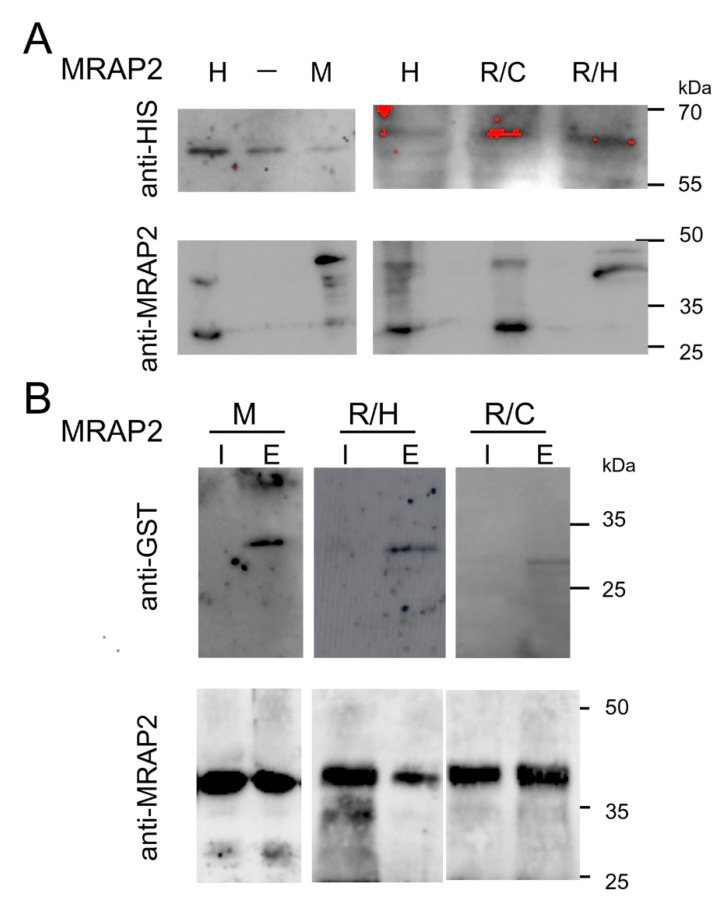
Interaction of MRAP2 isoforms with PKR2. (**A**) Membrane proteins from yeast co-expressing MRAP2 isoforms with PKR2 were co-precipitated using Ni-NTA His-bind resin and resolved by 12% SDS-PAGE. The immunoblots were probed with anti-His and anti-MRAP2 antibodies. Mouse MRAP2, M; human MRAP2, H; MRAP2 R125H, R/H; R125C MRAP2, R/C. (**B**) GST pull-down experiments. The GST fusion protein of PKR2-NT was used to pull down MRAP2 isoforms. Mouse MRAP2, M; R125H mutant, R/H; R125C mutant, R/C. Input and solutions obtained by elution were analyzed by western blotting with anti-GST and anti-MRAP2. I, input; E, eluate.

We expressed mouse MRAP2 and human R125H and R125C MRAP2 mutants with PKR2 terminal tagged His (PKR2-His) in *S. cerevisiae* Cy12946. Total membrane proteins were purified by Ni-NTA chromatography and subjected to Western blotting analysis. As shown in Figure 3A, MRAP2 isoforms bind to PKR2, as indicated by the signal obtained with the anti-His and anti-MRAP2 antibodies. To confirm the interaction, we performed a GST (glutathione S-transferase) pull-down assay with a protein obtained by fusing the N-terminal region of PKR2 with GST and MRAP2 isoforms produced in yeast. Thus, lysates of *E. coli* cells expressing GST fusion of the N-terminal region of PKR2 were loaded on glutathione–Sepharose columns and then incubated with mouse MRAP2 and the human MRAP2 mutants R125H and R125C. As shown in Figure 3B, the N-terminal region of PKR2 was able to pull down the different MRAP2 isoforms. Importantly, GST alone was not able to pull down MRAP2 (data not shown).

### 2.3. Expression and Biochemical Analysis of C Terminal Region of Mouse MRAP2 and MRAP2 Mutants in E. coli

**Figure 4:**
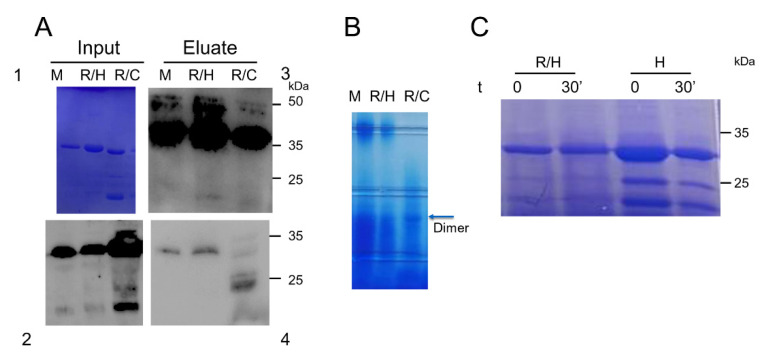
Biochemical analysis of CT-MRAP2 domain. (**A**) GST pull-down experiments. The CT-MRAP2-GST fusion proteins were used to pull down CT-domains of mouse MRAP2, M, and MRAP2 mutant_domains, R/H and R/C. The input was analyzed by SDS-PAGE (Panel 1) and western blotting analysis with antibody anti-HIS (Panel 2). Solutions obtained by elution were analyzed by western blotting with anti-GST (Panel 3) and anti-HIS (Panel 4) antibodies. (**B**) Blue native PAGE of mouse MRAP2-CT, M and MRAP2 mutants-CT -domains, R/H and R/C expressed in *E*. *coli*. (**C**) SDS-PAGE analysis of human MRAP2-CT, H, and R/H-CT, R/H limited proteolysis time course.

The C-terminal region of MRAP2 encoded by the fourth exon (MRAP2-CT) retains the ability to dimerize, and the protein migrates as a dimer even under denaturing conditions, as described previously [5]. We performed a GST pull-down experiment to determine the ability of mouse MRAP2 and the MRAP2 mutants to form dimers. Lysate from cells expressing mouse MRAP2, R125H, and R125C carboxy terminal domain GST fusion proteins were incubated with glutathione–Sepharose resin in the presence of the purified His-tagged MRAP2 C terminal domains, denominated as mMRAP2-CT, R/H-CT, and R/C-CT. As shown in Figure 4A, mMRAP2-GST, R/H-GST, and R/C-GST were able to pull down MRAP2 domains, demonstrating the ability of the C terminal domain of MRAP2 mutants to form homodimers. In contrast, GST alone was not able to pull down MRAP2.

These results were confirmed by blue native PAGE. Based on the molecular weights of the bands, we were able to detect the presence of structures that are most likely dimers and oligomers, although the prediction of molecular weights in blue native PAGE is approximate (Figure 4B).

To analyze the conformational change induced by the substitution of arginine 125 with histidine, we compared proteolysis analysis of R/H-CT with human MRAP2-CT wt. It was clear that the effects on proteolysis were significantly different (Figure 4C). 

## 3. Discussion

MRAP2 is a glycosylated protein with a single transmembrane domain with no predicted signal peptide. MRAP2 is specifically expressed in the brain, where it regulates the maturation, transport, and signaling of GPCRs involved in energy balance and food intake. Mrap2-deficient mice develop severe obesity, and many MRAP2 variants have been found in severely obese human patients [11,12,14]. Comparisons between MRAP and MRAP2 show that the N-terminal and transmembrane domains have a high degree of homology, whereas the C-terminal region diverges. The C-terminal domain is characterized by a diffuse and intrinsically disordered structure and by a small region with a distinct secondary structure, namely, three alpha-helix and five beta strands [15]. For proteins involved in the mechanism of signal transduction, the presence of an intrinsically disordered region is functionally important because the flexibility allows them to interact with multiple protein partners and adopt a partner-specific ordered structure only after the interaction [16]. The C-terminal region of MRAP2 contains an arginine residue at position 125 that is essential for MRAP2 function; mutation of this residue causes a highly obese phenotype in humans [11,12,14]. We characterized the obesity-associated MRAP2 mutants R125H and R125C in terms of regulation of PKR2 activity. R125H reduces PK2-induced ERK activation more effectively than R125C and MRAP2 wt. In the presence of MRAP2 mutants, a change in PKR2 glycosylation is observed, as in the presence of MRAP2 wt [17]. MRAP2 has the ability to form a unique topology in eukaryotes characterized by a dual-oriented dimer structure. Our results defined the minimal C-terminal domain region of MRAP2 as sufficient to ensure dimer formation [5]. Other MRAP2 regions were identified as important for MRAP2 dimer formation, particularly the N-terminal region and the transmembrane domain [18,19]. These different results are likely due to the complexity of the structure that may show a propensity for dimer formation, with dual orientations and a tendency to form oligomeric structures as well. It is likely that the protein can adopt two alternative conformations in the dimeric and monomeric forms that are in equilibrium. Biochemical analysis showed that the arginine residue at position 125 modulates the activity and dimerization ability of the protein. In particular, our results suggest that mutation of arginine at position 125 in histidine could stabilize the conformation in the dimer and shift the equilibrium toward the dimeric form. Previously, in silico analysis demonstrated that the arginine 125 mutation leads to a change in protein conformation [15]. Our result suggests that this conformational change leads to a greater propensity of the mutant protein to form dimers. 

In this paper, we examined the mouse MRAP2 protein, demonstrating that it has the same characteristics highlighted for the R125H mutant.

The mouse MRAP2 homolog, which naturally has this substitution, is able to inhibit human PKR2 receptor signaling more effectively than the human isoform [3]. Mouse MRAP2 differentially regulates melanocortin receptor 2; it was only able to support a weak cAMP response to ACTH [18], whereas human MRAP2 elicited a strong response [20]. In vivo analysis of mouse MRAP2 by electrophoresis revealed a protein with a predicted molecular weight of 47.3 kDa, presumably corresponding to a dimeric MRAP2 structure resistant to the action of SDS, reducing agents, and heat. In contrast, human MRAP2 migrates as a monomeric form, and two molecular species with sizes of 27 and 29 kDa were detected, representing the deglycosylated and glycosylated protein, respectively [21]. Studying the molecular details of MRAP2 structure is important to understanding the mechanism by which MRAP2 regulates G protein-coupled receptors and can help to elucidate the pathways involved in metabolic dysfunction. These results are particularly interesting because they could aid in the development of new drugs that favor the dissociation of the MRAP2-PKR2 complex specifically blocking one signaling pathway.

## 4. Materials and Methods

### 4.1. Expression of Constructs

The procedure for obtaining pYESC3-PKR2 has been described previously [22]. For constitutive expression of mouse MRAP2 MYC-DDk-tagged in mammalian cell lines, plasmid OMu00763 was purchased from Genescript, Piscataway, NJ, USA.

For the expression of mouse MRAP2 in *S. cerevisiae*, the p413 mouse-MRAP2 plasmid was constructed. The cDNA of mouse MRAP2 was amplified by PCR using the plasmid OMu00763 as a template, with the oligonucleotides MRAP2 BamHI up and MRAP2 EcoRI dw and cloned into p413 digested with BamHI and EcoRI.

For constitutive expression of MYC-DDk-tagged human MRAP2 in mammalian cell lines, RC203668 plasmid was purchased from Origene (Fisher Scientific, Waltham, MA, USA).

The p413 humanMRAP2 plasmid previously described in [5] was used as a template to construct the R125H and R125C MRAP2 mutants with the QuikChange II Site-Directed Mutagenesis Kit (Agilent, Santa Clara, CA, USA), using oligonucleotides R125H for and R125H rev for substitution of arginine by histidine and primers R125C for and R125C rev for substitution of arginine by cysteine, respectively. The obtained plasmids were designated p413 R125H and p413 R125C. The fragments obtained by digestion of p413-R125H and p413-R125C with BamHI and EcoRI were cloned into pcDNA to obtain pcDNA-R125H pcDNA-R125C.

For expression of the carboxy-terminal domain of MRAP2 in *E. coli*, the DNA fragment coding for the C-terminal domain was amplified by PCR with gene-specific primers T70 BamHI and MRAP2 EcoRI dw, using as templates R125H-pcDNA, R125C-pcDNA, and OMu00763, respectively, and cloned into pBluescript to obtain R125H-CT pBS and R125C-CTpBS and mMRAP2-CTpBS. The resulting plasmids were digested with BamHI and EcoRI, and the obtained fragments were cloned into pET28a and PGEX-2T. All oligonucleotides used for the plasmid constructions are listed in Table 1.

### 4.2. CHO-R2 Cell Culture, Transfection, and Stimulation

CHO (Chinese Hamster Ovary) cells, which stably express human PKR2, were plated at a density of 4 × 10^5^ per well in six-well plates and grown in Dulbecco’s Modified Eagle Medium/Nutrient mixture F-12 Ham (DMEM/F12) containing 100 U/mL penicillin/streptomycin, 10% fetal bovine serum (FBS), 2 mM L-glutamine, and G418 200 ng/mL (Sigma-Aldrich, Milan, Italy) at 37 °C and 5% CO_2_. When the cells reached 70–80% confluence, transient transfection with plasmid DNA of mouse MRAP2, human MRAP2, and the R125H MRAP2 and R125C MRAP2 mutants was performed using Lipofectamine 2000 (Invitrogen, Life Technologies, Carlsbad, CA, USA) according to the manufacturer’s instructions. Briefly, cells were incubated with the lipofectamine–plasmid DNA complexes for 24 h at 37 °C and 5% CO_2_. Lipofectamine–plasmid DNA complexes were obtained by incubating 2 µg of plasmid DNA of mouse MRAP2, human MRAP2, and R125H the MRAP2 and R125C MRAP2 mutants with Lipofectamine 2000 diluted in Opti-MEM (Invitrogen) for 20 min at room temperature. After transfection, cells were deprived of serum and stimulated with PK2 100 nM for 10 min and 1 h at 37 °C and 5% CO_2_. At the end of the incubation period, cells were lysed in protein extraction buffer. Proteins were then quantified by the Bradford method and used for Western blot analysis.

### 4.3. Western Blot Assay

Western blot was performed as reported in Fullone et al. 2022 [5]. The following primary antibodies were used at a dilution of 1:1000 in the blocking solution: rabbit anti-ERK (Santa Cruz Biotechnology, Dallas, TX, USA, sc-153), mouse anti-p-ERK (Cell Signaling Technology, Danvers, MA, USA, 9106S), monoclonal anti polyhistidine peroxidase (Sigma-Aldrich, MERCK, Darmstadt, Germany A7058), rabbit anti-MRAP2 polyclonal antibody (Invitrogen-Thermo Fisher Scientific, Waltham, MA, USA, PA5-113283), mouse anti-PKR2 polyclonal antibody (Santa Cruz Biotechnology, Dallas, TX, USA, sc-365696), mouse anti-STAT3, and rabbit anti-pSTAT3 (Tyr705) (1:1000, Invitrogen, Thermo Fisher Scientific, Waltham, MA, USA). After extensive washing with T-TBS, membranes were incubated with the appropriate IgG HRP-linked secondary antibody for 1 h at room temperature. The immunoreactive signals were visualized using an enhanced chemiluminescence system.

### 4.4. Yeast Culture and Transformation

Transformation of the Cy12946 *S. cerevisiae* strain used for co-expression of PKR2, mouse MRAP2, and the R125H-MRAP2 and R125C-MRAP2 mutants was performed according to the procedure described in Fullone et al. 2022 [5]. Transformed yeast cells were grown at 30 °C to mid-log phase in appropriate selection media containing 2% raffinose as the sole carbon source. Protein expression was induced by the addition of galactose at a final concentration of 20 g/L.

### 4.5. Preparation of Yeast Membrane Proteins and Co-Precipitation

Solubilized membrane fractions co-expressing PKR2, mouse MRAP2, and the R125H-MRAP2 and R125C-MRAP2 mutants were co-precipitated by metal chelation chromatography using Ni-NTA His-bind resin after batch purification under native conditions according to the manufacturer’s instructions (Novagen, Sigma-Aldrich, Milan, Italy). The eluates were fractionated by SDS-PAGE on a 12% gel and transferred to a polyvinylidene difluoride membrane. The membrane was coated with a monoclonal anti-His antibody conjugated to horseradish peroxidase (1:5000) and with a polyclonal anti-MRAP2 antibody.

### 4.6. Glutathione S-Transferase (GST) Pull-Down

The R2-GST proteins were obtained by fusion of the 69 amino acids of the amino terminus of PKR2 with GST [8]. mMRAP2-CT-GST, R/H-CT-GST, or R/C-CT-GST were obtained by fusing the C-terminal domain of MRAP2 with GST. GST pull-down was performed as reported in Lattanzi et al., 2021 [8]. Briefly, 50 µL of a slurry of glutathione–Sepharose beads equilibrated in buffer A (PBS, 1% Nonidet P 40, 1 mM EDTA) supplemented with protease inhibitor were incubated with 2 mL lysate of R2-GST, mMRAP2-CT-GST, R/H-CT-GST, or R/C-CT-GST for 1 h at 4 °C with constant stirring. Beads with bound GST-fusion proteins were collected by centrifugation and washed extensively in buffer A. Then, GST fusion proteins bound beads were incubated overnight at 4 °C with total membrane extracted from yeast expressing mouse MRAP2 and the R125H and R125C MRAP2 mutants or with His-tagged mouse MRAP2-CT, R/H-CT, and R/C-CT expressed and purified in *E. coli*, respectively. Beads were washed as described above and bound proteins were eluted with GSH according to the procedure of GE Healthcare and analyzed by Western blotting with anti-MRAP2 antibodies or anti-HIS antibodies as described in Section 4.3

### 4.7. Blue Native-PAGE

Blue native-PAGE of purified MRAP2-CT, R/H-CT, R/C-CT was performed on NativePAGE Novex 3% to 16% Bis-Tris gels (Life Technologies—Thermo Fisher Scientific, Waltham, MA, USA) as described in Fullone et 2022 [5] using the buffers and conditions specified by the manufacturer and described in [23]; 20 µg of each protein sample was analyzed.

### 4.8. Limited Proteolysis Experiments

Protein samples (5 µg) were incubated with trypsin from bovine pancreas (0.002 µg/L; Sigma-Aldrich, Milan, Italy) at 20 °C in 50 mM NaHepes, pH 8.0, containing 300 mM NaCl and glycerol 5% (*v*/*v*). At specific time intervals, the proteolytic reaction was stopped by the addition of SDS and samples were boiled. Samples were then analyzed by SDS/PAGE.

### 4.9. Data Analysis

Data were analyzed with GraphPad Prism 6 for Windows using One-Way ANOVA followed by Tukey’s post hoc test. Results are expressed as mean ± SEM and differences were considered significant at *p* < 0.05.

## Figures and Tables

**Table 1:** Oligonucleotides used in this study.

Oligonucleotide	Sequence
MRAP2 *BamH*I up	5′-AAG GAT CCA TGTCCGCCCAGAGG-3′
MRAP2*EcoR*I dw	5′-AAGAATTCTTAAACCTTATCGTC-3′
T70 BamHI	5′-GGATCCACCAAGACAGGAGCCCCA-3′
R125H for	5′-GAGGTGGAACACTTGGACAGAGCCAAAGCATGT-3′
R125H rev	5′-ACATGCTTTGGCTCTGTCCAAGTGTTCCACCTC-3′
R125C for	5′-GAGGTGGAATGCTTGGACAGAGCCAAAGCATGT-3′
R125H rev	5′-ACATGCTTTGGCTCTGTCCAAGCATTCCACCTC-3′

## Data Availability

All the data presented in this study are available upon request from the corresponding author.
